# The steps that young people and suicide prevention professionals think the social media industry and policymakers should take to improve online safety. A nested cross-sectional study within a Delphi consensus approach

**DOI:** 10.3389/frcha.2023.1274263

**Published:** 2023-12-15

**Authors:** Jo Robinson, Pinar Thorn, Samuel McKay, Hannah Richards, Rikki Battersby-Coulter, Michelle Lamblin, Laura Hemming, Louise La Sala

**Affiliations:** ^1^ Suicide Prevention, Orygen, Parkville, VIC, Australia; ^2^ Centre for Youth Mental Health, The University of Melbourne, Parkville, VIC, Australia; ^3^ Violet Vines Marshman Research Centre, La Trobe Rural Health School, La Trobe University, Flora Hill, VIC, Australia

**Keywords:** suicide, self-harm, social media, young people, survey

## Abstract

**Introduction:**

Concerns exist about the relationship between social media and youth self-harm and suicide. Study aims were to examine the extent to which young people and suicide prevention professionals agreed on: (1) the utility of actions that social media companies currently take in response to self-harm and suicide-related content; and (2) further steps that the social media industry and policymakers could take to improve online safety.

**Methods:**

This was a cross-sectional survey study nested within a larger Delphi expert consensus study. A systematic search of peer-reviewed and grey literature and roundtables with social media companies, policymakers, and young people informed the questionnaire development. Two expert panels were developed to participate in the overarching Delphi study, one of young people and one of suicide prevention experts; of them 43 young people and 23 professionals participated in the current study. The proportion of participants “strongly agreeing”, “somewhat agreeing”, “neither agreeing nor disagreeing”, and “somewhat disagreeing” or “strongly disagreeing” for each item were calculated; items that achieved =>80% of agreement from both panels were strongly endorsed.

**Results:**

There was limited consensus across the two groups regarding the utility of the safety strategies currently employed by companies. However, both groups largely agreed that self-harm and suicide-related content should be restricted. Both groups also agreed that companies should have clear policies covering content promoting self-harm or suicide, graphic depictions of self-harm or suicide, and games, pacts and hoaxes. There was moderate agreement that companies should use artificial intelligence to send resources to users at risk. Just over half of professionals and just under half of young people agreed that social media companies should be regulated by government. There was strong support for governments to require schools to educate students on safe online communication. There was also strong support for international collaboration to better coordinate efforts.

**Discussion:**

Study findings reflect the complexity associated with trying to minimise the risks of communicating online about self-harm or suicide whilst capitalising on the benefits. However, a clear message was the need for better collaboration between policymakers and the social media industry and between government and its international counterparts

## Introduction

Suicide is the fourth leading cause of death among young people worldwide (1), and the leading cause of death in many countries including Australia (2–4). Self-harm (defined here as an act of deliberate self-injury or self-poisoning, irrespective of motive or suicidal intent) (5) is also prevalent among young people (6, 7) and is a key risk factor for future suicide (8).

Although the reasons for self-harm and suicide are complex, there is concern about the role social media plays in introducing or exacerbating psychological distress among young people (9, 10). There are also fears regarding the potential for certain types of content, for example, graphic imagery and livestreams of self-harm and suicidal acts, to cause distress and lead to imitative behaviour among others (11). However, social media is popular among young people and our work has identified several potential benefits (12). For example, it allows young people to feel a sense of community, to seek help for themselves and to help others, and to grieve for people who have died by suicide. Additional benefits include its accessibility, non-stigmatizing nature, and capacity to deliver highly personalized content directly to a user's feed (12, 13). While this work highlights the potential for social media to be a useful tool for suicide prevention, we need to identify ways to minimize the risks associated with social media without simultaneously diminishing the benefits.

Potential levers for creating and maintaining a safe and healthy online environment for users include educational approaches, whereby users are provided with the information to keep themselves and their peers safe online; policy approaches, whereby governments develop and implement legislation to support online safety; and industry self-regulation, whereby the social media industry takes responsibility for maximising safety on its platforms (14).

Progress is being made in each of these areas. For example, the #chatsafe program, comprising evidence-informed guidelines plus social media content, is an example of an educational approach that has been shown to better equip young people to communicate safely online about suicide (12, 15, 16). Although important, many would argue that the onus should not be placed on young people alone to keep themselves safe online. Therefore, additional approaches are required.

Governments in several countries have taken steps towards developing policies designed to regulate the social media industry. For example, the United Kingdom (UK) (17), Australia (18), and the United States of America (USA) (19, 20) have all introduced legislation designed to regulate the social media industry. Though these are broad-brush approaches, they provide guidance to platforms on operating safely and impose fines and penalties on companies that do not comply. However, the rapid evolution of social media, together with the lengthy process of passing legislation, means we cannot rely on legislation alone. Further, restrictions imposed in one country does not necessarily prevent distressing information spreading among young people elsewhere.

Finally, the social media industry has also taken steps to improve user safety on their platforms. For example, many platforms have appointed safety advisory boards and developed safety functions that help users control the type of content they view and interact with (14). However, little is known about either the acceptability or perceived utility of these measures among young people. To the best of our knowledge, only one study, conducted by The Samaritans in the UK has examined this. This study was a cross sectional survey of 5,294 people living in the UK (87% of whom had a history of self-harm) and found that more than 75% of the sample had viewed self-harm content online before the age of 14 years (11). Of those who encountered self-harm and suicide-related content, 83% reported that they had not intentionally searched for it; many reported that it worsened their mood, and over three quarters reported that they self-harmed in similar, or a more severe way, after exposure to the content. Despite mixed findings related to the efficacy of trigger warnings (21), study participants reported that a specific content warning (as opposed to a generic one) would have been helpful. Overall, participants were largely in favour of having more control over the content that they see, such as the ability to mute certain types of content or block other users.

Each of these approaches are in their infancy, and it is unlikely that one approach alone will be sufficient to create and maintain safe online environments. Further, questions remain as to how helpful young people and suicide prevention professionals consider existing approaches to be, and what additional steps they believe could be taken by the social media industry and policymakers.

### Aims

The aim of this study was to examine what young people and suicide prevention professionals believed that the social media industry and policymakers should do to create and maintain safer online environments in relation to communication about self-harm and suicide. An additional aim was to seek the views of participants on some of the actions that social media companies are already taking in response to self-harm and suicide-related content.

## Methods

### Study design

This was a cross sectional survey. It was nested within a larger Delphi expert consensus study (reported elsewhere) that was conducted to update and expand the original #chatsafe guidelines (16) and involved: (1) a systematic search of peer-reviewed and grey literature; (2) roundtables with social media companies, policymakers, and young people; (3) questionnaire development; (4) expert panel formation; (5) data collection and analysis; and (6) guideline development.

The study received approval from The University of Melbourne Human Research Ethics Committee (ID: 22728).

### Systematic search of the literature

Sources published in the peer-reviewed or grey literature were eligible for inclusion if they focused on: (1) self-harm or suicide; (2) social media or other online environments; and (3) the nature of online communication about self-harm or suicide. Peer-reviewed articles had to be written in English, French, Spanish, or Russian (i.e., the languages spoken by the research team) to be included. Grey literature had to be written in English.

For the peer-reviewed literature search, CINAHL, EMBASE, ERIC, Medline, PsycINFO, and Scopus were searched for studies published between 1 January 2000 and 4 November 2021. The grey literature search involved three components. First, the following databases were searched: APAIS-Health, Australian Policy Online, and ProQuest Dissertations and Theses Global (PQDT). Second, the first ten pages (i.e., up to the first 100 results) of google.com, google.com.au, google.ca, google.co.nz, and google.co.uk were searched. Finally, the “help centers” or equivalent of Ask FM, Clubhouse, Deviant Art, Discord, Facebook, Instagram, Pinterest, Quora, Snapchat, TikTok, Tumblr, Twitch, Twitter, WhatsApp, and YouTube were searched and screened. The full search strategy has been published elsewhere (22).

### Round table consultations

Six roundtable consultations were conducted between June and August 2022: three with social media companies (*n* = 7), two with policymakers from the Australian federal government (*n* = 14), and one with young people (*n* = 7). Discussions focused on: (1) the challenges associated with online communication about self-harm and suicide; (2) what more (if anything) social media platforms and policymakers could be doing to keep young people safe online; (3) the extent to which online safety is the responsibility of government, platforms and/or individuals. Each session was audio recorded, transcribed and potential action items were extracted for inclusion in the questionnaire—see below.

### Questionnaire development

Statements extracted from 149 peer-reviewed articles, 52 grey literature sources, and the roundtables formed the basis of the current questionnaire. Participants were informed that these items were designed to identify what actions participants thought that the social media industry and policymakers could be doing to improve online safety.

There were 158 survey non-forced response items in this section of the survey (See Supplementary File 1). Examples include: “Social media companies should provide clear policies on safe and unsafe online behaviour in relation to suicide / self-harm”; “Government should create a rating system of social media companies against a set of safety standards”. Participants were asked to rate the extent to which they agreed with items on a 5-point Likert scale, with 1 being strongly disagree and 5 being strongly agree. Participants were also asked to indicate how social media companies should manage graphic or potentially unsafe content. For these 10-items participants responded using one of the following five response options: “remove”, “shadow ban”, “allow users to view content but disable interactions”, “restrict”, and “unsure”.

### Participants and recruitment

Two expert panels were recruited. One comprised suicide prevention professionals identified via the literature search and one comprised young people recruited via social media advertisements.

Professionals were invited to take part via email and were eligible for inclusion if they were: (1) aged at least 18 years; (2) an expert on self-harm or suicide (e.g., research, teach, or treat self-harm or suicide; and had published research on self-harm or suicide and social media, or contributed to guidelines on communication about self-harm or suicide); and (3) were proficient in English. Young people were recruited via Instagram advertisements and were eligible for inclusion if they: (1) were aged between 15 and 25 years inclusive; (2) lived in Australia; (3) were proficient in English; and (4) had seen, communicated about, or wanted to communicate online about self-harm or suicide.

### Data analysis

Data were analysed in Microsoft Excel. The proportion of participants “strongly agreeing”, “somewhat agreeing”, “neither agreeing nor disagreeing”, and “somewhat disagreeing” or “strongly disagreeing” for each item were calculated for both professionals and young people separately. We used a similar approach to the Delphi consensus study in the current analysis. Specifically, items that achieved =>80% of agreement from both panels (either “somewhat” or “strongly”) were considered to be strongly endorsed and steps that should be taken, items that received an agreement level (either “somewhat” or “strongly”) between 70 and 79.99% were considered moderately endorsed and steps worth considering. If an item received agreement (either “somewhat” or “strongly”) from less than 70% of either panel, it was considered to have low endorsement and to unnecessary at this time. In cases where there was large discrepancy in endorsement (e.g., 50% of one group and 70% of the other group agreed with the item) the lowest score was taken as the level of endorsement. This approach allowed us to identify key actions that were considered a priority for social media companies and policymakers by both professionals and young people. For the 10 items that asked participants to rate how social media companies should manage graphic or potentially unsafe content, the proportion of agreement for each of the five response options (e.g., “remove”, “shadow ban”, “allow users to view content but disable interactions”, “restrict”, and “unsure”) was reported and any major discrepancies between panels were noted in the results.

## Results

### Sample characteristics

Forty-three young people responded to this survey. The mean age of youth participants was 21.30 years (*SD* = 2.54, range = 17–25). Seventy-two per cent identified as female, 20.9% as transgender or gender diverse, and 7.0% as male. Just under one half identified as LGBTIQA + (48.8%), and almost one-quarter came from a culturally or linguistically diverse background (23.3%). Most had their own lived experience of self-harm or suicide (81.4%), and/or had supported someone who was self-harming or suicidal (65.1%); 16.3% were bereaved by suicide.

Twenty-three professionals responded to the survey. They included PhD students (17.4%), postdoctoral researchers or lecturers (26.1%), professorial staff (43.5%); 8.7% were in roles such as research advisors or funders. One participant did not report their role. Almost one fifth also worked as clinicians (17.4%). They resided in a variety of countries including Australia (26.1%), UK (17.39%), USA (13.0%), and 4.4% each from Austria, Canada, China, Estonia, France, Germany, Ghana, Hong Kong, New Zealand, South Africa, and Spain.

### Key findings

The results presented below are broken down as follows: (1) actions that the social media industry should take in terms of enhancing safety policies, responding to self-harm and suicide-related content, and staffing; and (2) actions that policymakers should take, including better industry regulation, educational programs, and future collaboration and investment.

#### Actions the social media industry should take policies, responsibility and reporting

Table 1 presents the views of both young people and suicide prevention professionals with regards to the development and implementation of safety policies, where responsibility for online safety lies, and reporting. Both professionals and young people agreed that companies should have clear and accessible policies that cover content promoting or encouraging self-harm or suicide, graphic depictions of self-harm or suicide, and games, pacts and hoaxes. Participants agreed that policies should be developed in collaboration with platform users (both with and without lived experience), suicide prevention and mental health professionals, legal and communications professionals, other industry professionals and policymakers. There was also strong agreement that social media companies hold responsibility for the content posted on their platforms and that they should maximise user agency by enabling users to control the type of content that they see.

**Table 1:** Current strategies employed by platforms.

Social media companies should	Professionals						Young people					
	N	Remove (i.e, take down/delete)	Shadow ban (i.e., remove from public view. The poster will still see the post)	Allow users to view content, but disable interactions (i.e., users can see the post but cannot share, comment, like or react)	Restrict (e.g., add an age restriction, content warning, or blur)	Unsure	N	Remove (i.e, take down/delete)	Shadow ban (i.e., remove from public view. The poster will still see the post)	Allow users to view content, but disable interactions (i.e., users can see the post but cannot share, comment, like or react)	Restrict (e.g., add an age restriction, content warning, or blur)	Unsure
Any images depicting suicide / self-ham	21	38.10%	33.33%	0.00%	19.05%	9.52%	43	34.88%	23.26%	2.33%	30.23%	9.30%
Images of new suicide / self-harm wounds and injuries	21	38.10%	28.57%	0.00%	28.57%	4.76%	43	51.16%	23.26%	2.33%	18.60%	4.65%
Images of healed suicide / self-harm injuries	21	19.05%	19.05%	0.00%	61.90%	0.00%	43	2.33%	9.30%	4.65%	74.42%	9.30%
Artistic suicide / self-harm images (e.g., tattoos)	21	4.76%	23.81%	0.00%	47.62%	23.81%	43	6.98%	2.33%	9.30%	53.49%	27.91%
Images of suicide / self-harm methods and locations	21	57.14%	28.57%	0.00%	4.76%	9.52%	43	62.79%	11.63%	0.00%	18.60%	6.98%
Hashtags related to suicide / self-harm	21	4.76%	23.81%	0.00%	47.62%	23.81%	43	9.30%	9.30%	4.65%	55.81%	20.93%
Livestreams of suicide / self-harm	21	66.67%	19.05%	0.00%	0.00%	14.29%	42	71.43%	14.29%	0.00%	9.52%	4.76%
Responses to livestreams of suicide / self-harm	21	52.38%	23.81%	4.76%	0.00%	19.05%	43	44.19%	18.60%	4.65%	18.60%	13.95%
Videos of the lead up to or process of suicide	21	71.43%	23.81%	0.00%	0.00%	4.76%	43	76.74%	13.95%	2.33%	4.65%	2.33%
Videos of suicide rescue footage	21	28.57%	28.57%	0.00%	23.81%	19.05%	43	34.88%	13.95%	4.65%	34.88%	11.63%
Graphic descriptions of self-harm / suicide	21	42.86%	42.86%	0.00%	4.76%	9.52%	43	55.81%	9.30%	0.00%	32.56%	2.33%
Suicide notes	21	33.33%	38.10%	0.00%	19.05%	9.52%	43	27.91%	13.95%	0.00%	32.56%	25.58%
Fictional suicide / self-harm content (e.g., illustrations, animations, memes, video games)	21	4.76%	23.81%	0.00%	47.62%	23.81%	43	16.28%	2.33%	11.63%	48.84%	20.93%
Suicide hoaxes	21	80.95%	9.52%	0.00%	4.76%	4.76%	43	79.07%	4.65%	4.65%	4.65%	6.98%
Suicide games and challenges	21	80.95%	19.05%	0.00%	0.00%	0.00%	43	90.70%	6.98%	0.00%	2.33%	0.00%
Educational suicide / self-harm content	19	5.26%	5.26%	31.58%	36.84%	21.05%	43	4.65%	4.65%	11.63%	65.12%	13.95%
Misinformation about suicide / self-harm	21	66.67%	23.81%	0.00%	4.76%	4.76%	43	81.40%	6.98%	0.00%	6.98%	4.65%
Content that identifies and shares details of those who have engaged in suicide / self-harm (doxing)	21	71.43%	9.52%	4.76%	4.76%	9.52%	43	79.07%	4.65%	6.98%	4.65%	4.65%
Accounts dedicated to suicide / self-harm of any nature	21	14.29%	19.05%	0.00%	38.10%	28.57%	43	39.53%	2.33%	6.98%	34.88%	16.28%
Accounts that are pro-suicide / self-harm	21	66.67%	9.52%	0.00%	4.76%	19.05%	43	90.70%	6.98%	0.00%	2.33%	0.00%
Accounts that support understanding, reduction, cessation of suicide / self-harm	20	0.00%	0.00%	15.00%	45.00%	40.00%	43	0.00%	4.65%	11.63%	58.14%	25.58%

In terms of reporting, participants believed companies should promote a culture of reporting content that violates their policies, should review all self-harm or suicide related reports, and if content is removed they should explain to users why this is the case. There was strong agreement that companies should expand reporting categories to cover a broader range of content.

#### Managing and responding to self-harm and suicide-related content

Table 2 presents the views of participants with regards to the ways in which platforms should manage and respond to self-harm and suicide-related content.

**Table 2:** Policies, responsibility, and reporting.

Question	Professionals						Young people					
	N	Strongly Disagree %	Somewhat Disagree %	Neither Agree nor Disagree %	Somewhat Agree %	Strongly Agree %	N	Strongly Disagree %	Somewhat Disagree %	Neither Agree nor Disagree %	Somewhat Agree %	Strongly Agree %
Companies should provide clear policies on safe and unsafe online behaviour in relation to suicide/self-harm	23	0.00	0.00%	0.00%	13.04%	86.96%	42	0.00%	0.00%	0.00%	19.05%	80.95%
Companies should outline how they will respond to unsafe content and what actions will be taken against a user if they do not comply with safety policies	23	0.00%	0.00%	0.00%	17.39%	82.61%	42	0.00%	0.00%	0.00%	4.76%	95.24%
Companies should make policies easily accessible and visible on their platforms	23	0.00%	0.00%	0.00%	4.35%	95.65%	41	0.00%	0.00%	0.00%	7.32%	92.68%
If someone dies by suicide, the company should contact the next of kin to ask whether they wish to memorialise the person's profile	23	8.70%	17.39%	34.78%	26.09%	13.04%	42	0.00%	7.14%	4.76%	38.10%	50.00%
Companies should specifically develop policies on												
Content that promotes or encourages suicide/self-harm	23	0.00%	0.00%	0.00%	8.70%	91.30%	43	6.98%	0.00%	0.00%	16.28%	76.74%
Content that contains graphic descriptions or visual depictions of suicide/self-harm	23	0.00%	0.00%	0.00%	8.70%	91.30%	43	6.98%	0.00%	0.00%	20.93%	72.09%
Content that details methods of, or instructions about how to engage in suicide/self-harm	23	0.00%	0.00%	0.00%	4.35%	95.65%	43	2.33%	0.00%	4.65%	13.95%	79.07%
Suicide pacts, challenges, games, and hoaxes	23	0.00%	0.00%	0.00%	0.00%	100.00%	43	6.98%	0.00%	2.33%	13.95%	76.74%
Mocking, doxing (publishing private and identifying information without consent), or bullying/harassing of users who have self-harmed, attempted suicide, or died by suicide	23	0.00%	0.00%	0.00%	0.00%	100.00%	43	2.33%	0.00%	2.33%	16.28%	79.07%
Social media companies should work collaboratively with the following groups to develop policies and educational campaigns and resources												
Users regardless of living or lived experience of suicide/self-harm (i.e., young people)	23	0.00%	0.00%	21.74%	47.83%	30.43%	43	2.33%	4.65%	2.33%	34.88%	55.81%
Users with lived or living experience of suicide/self-harm	23	0.00%	0.00%	8.70%	34.78%	56.52%	43	0.00%	0.00%	2.33%	11.63%	86.05%
Suicide prevention experts (e.g., academic researchers)	22	0.00%	0.00%	0.00%	13.64%	86.36%	43	0.00%	0.00%	0.00%	9.30%	90.70%
Mental health professionals	23	0.00%	0.00%	4.35%	21.74%	73.91%	43	0.00%	0.00%	2.33%	9.30%	88.37%
Media and communications professionals	23	0.00%	0.00%	4.35%	39.13%	56.52%	43	0.00%	2.33%	9.30%	32.56%	55.81%
Emergency service personnel	23	0.00%	0.00%	21.74%	43.48%	34.78%	43	0.00%	4.65%	6.98%	32.56%	55.81%
Legal experts	23	0.00%	0.00%	8.70%	43.48%	47.83%	43	2.33%	2.33%	11.63%	41.86%	41.86%
Parents/guardians	23	0.00%	4.35%	26.09%	43.48%	26.09%	43	2.33%	6.98%	18.60%	34.88%	37.21%
Teachers	23	0.00%	8.70%	39.13%	34.78%	17.39%	43	4.65%	6.98%	20.93%	27.91%	39.53%
Influencers	23	4.35%	8.70%	39.13%	30.43%	17.39%	43	16.28%	16.28%	16.28%	23.26%	27.91%
Search engines (e.g., Google)	23	0.00%	4.35%	30.43%	30.43%	34.78%	43	9.30%	13.95%	23.26%	27.91%	25.58%
Other social media companies	23	0.00%	4.35%	34.78%	26.09%	34.78%	43	6.98%	2.33%	16.28%	37.21%	37.21%
External support services such as helplines and counselling services	23	0.00%	0.00%	26.09%	34.78%	39.13%	43	0.00%	0.00%	2.33%	20.93%	76.74%
Policymakers/governments	23	0.00%	4.35%	13.04%	52.17%	30.43%	43	2.33%	4.65%	2.33%	37.21%	53.49%
Responsibility												
Companies are responsible for the content that is published in on their platforms	22	0.00%	0.00%	22.73%	59.09%	18.18%	43	0.00%	2.33%	9.30%	51.16%	37.21%
Companies and policymakers are both responsible for safety; however, the platform should own the bulk of the responsibility for safety of users	22	0.00%	4.55%	22.73%	54.55%	18.18%	43	0.00%	0.00%	6.98%	58.14%	34.88%
Companies should restrict underage users (>18 years) from exposure to suicide/self-harm content	22	9.09%	9.09%	27.27%	18.18%	36.36%	43	2.33%	11.63%	18.60%	23.26%	44.19%
Companies should maximise user agency by building in functions that enable users to filter specific types of content and decide what they want to see and what they do not want to see (e.g., suicide, self-harm etc.)	22	0.00%	0.00%	9.09%	36.36%	54.55%	43	0.00%	2.33%	6.98%	41.86%	48.84%
Reporting												
Policies should include how to report content	23	0.00%	0.00%	0.00%	4.35%	95.65%	43	0.00%	0.00%	0.00%	6.98%	93.02%
Companies should promote a culture of reporting and reduce stigma around this	22	0.00%	4.55%	18.18%	22.73%	54.55%	43	2.33%	2.33%	11.63%	23.26%	60.47%
Social media companies should provide users with step-by-step information on												
How to report harmful/ unsafe suicide/self-harm content to the platform	22	0.00%	0.00%	0.00%	9.09%	90.91%	43	0.00%	0.00%	0.00%	16.28%	83.72%
What happens after content is reported	22	0.00%	0.00%	0.00%	18.18%	81.82%	43	2.33%	0.00%	0.00%	16.28%	81.40%
If the user will be notified of who reported them	22	4.55%	4.55%	0.00%	9.09%	81.82%	43	0.00%	2.33%	0.00%	18.60%	79.07%
What is communicated to the user who was reported	22	4.55%	0.00%	4.55%	22.73%	68.18%	43	2.33%	0.00%	2.33%	20.93%	74.42%
Companies should review all suicide or self-harm related reports	22	0.00%	4.55%	0.00%	40.91%	54.55%	43	0.00%	2.33%	0.00%	23.26%	74.42%
Companies should prioritise user reports based on level of risk	22	0.00%	4.55%	27.27%	40.91%	27.27%	43	0.00%	9.30%	11.63%	30.23%	48.84%
Moderators should inform users about why their content has been removed	22	0.00%	0.00%	0.00%	36.36%	63.64%	43	0.00%	0.00%	0.00%	23.26%	76.74%
Companies should expand reporting categories to cover a broader range of unsafe behaviour online including suicide/self-harm	22	0.00%	0.00%	9.09%	45.45%	45.45%	42	2.38%	4.76%	0.00%	33.33%	59.52%
Companies should keep users who submit reports informed as to the general progress and actions taken because of the report.	19	5.26%	0.00%	31.58%	57.89%	5.26%	39	0.00%	12.82%	17.95%	30.77%	38.46%

There was moderate agreement that companies should use artificial intelligence (AI) to send helpful resources to users at risk, though less agreement on whether companies should use AI to intervene where risk was detected. There was strong agreement that companies should not allow self-harm or suicide content to appear in people's “suggested content”. There was also strong agreement that all companies should have a clearly accessible safety centre that contains evidence-based information and links to support services. Professionals and young people agreed that companies should restrict access to content that could be harmful to others and that membership of a platform should be paused if individuals repeatedly breach safety policies.

There was strong agreement that social media companies should provide content warnings for potentially harmful self-harm or suicide-related content and for content with self-harm or suicide-related hashtags. These should include information about why the content warning exists plus links to resources. There was weaker agreement with how livestreams of self-harm or suicidal acts should be managed, although >50% of both groups agreed that the stream should be removed by platforms immediately. There was strong agreement that platforms should send resources and links to support services to both the person posting the livestream and viewers, and the livestream should be reported to emergency services. However, there was low endorsement of platforms contacting law enforcement more broadly if a user appeared to be at risk of suicide. (37% of young people and 18% of professionals). Finally, there was strong agreement that platforms should actively promote helpful content such as messages that encourage help-seeking plus stories of hope and recovery.

Table 3 presents participants’ views on some of the safety strategies currently employed by social media companies. There was less consensus across the two groups for most of the items listed in Table 3, however for the most part the two groups did agree that access to the types of content listed should be restricted in some way. That said, >50% of both groups agreed that images of self-harm or suicide methods and locations should be removed, >60% agreed that livestreams should be removed and >70% agreed that videos depicting preparations for suicide should be removed. Around 80% of both groups agreed that content relating to suicide hoaxes, games and challenges should be removed. For several types of content, almost a quarter of participants stated they were unsure what social media companies should do.

**Table 3:** Responding to self-harm and suicide-related content.

Question	Professionals						Young people					
	N	Strongly Disagree %	Somewhat Disagree %	Neither Agree nor Disagree %	Somewhat Agree %	Strongly Agree %	N	Strongly Disagree %	Somewhat Disagree %	Neither Agree nor Disagree %	Somewhat Agree %	Strongly Agree %
Companies should utilise artificial intelligence (AI) to identify harmful mainstream media coverage of suicide/self-harm	22	0.00%	0.00%	22.73%	45.45%	31.82%	43	4.65%	4.65%	6.98%	53.49%	30.23%
Companies should utilise AI to identify users indicating risk of suicide/self-harm	22	4.55%	13.64%	18.18%	36.36%	27.27%	43	11.63%	11.63%	9.30%	44.19%	23.26%
Companies should use AI to send helpful information, resources, and links to support to users at risk	22	0.00%	9.09%	18.18%	40.91%	31.82%	43	6.98%	6.98%	11.63%	46.51%	27.91%
Companies should not use AI to detect risk and intervene in any way because it is unethical and should not be done	22	9.09%	45.45%	18.18%	18.18%	9.09%	43	9.30%	27.91%	25.58%	20.93%	16.28%
Companies should redirect search results for suicide/self-harm to more helpful content (e.g., content designed to educate or instil hope)	22	4.55%	4.55%	9.09%	45.45%	36.36%	43	6.98%	13.95%	20.93%	32.56%	25.58%
Companies should remove autocomplete searches for terms relating to suicide and self-harm methods	22	4.55%	0.00%	22.73%	27.27%	45.45%	43	2.33%	6.98%	16.28%	25.58%	48.84%
Companies should not allow harmful suicide/self-harm content to appear in “suggested content”	22	0.00%	0.00%	13.64%	18.18%	68.18%	43	2.33%	4.65%	4.65%	25.58%	62.79%
All social media platforms should have a safety centre	22	0.00%	0.00%	13.64%	31.82%	54.55%	43	0.00%	0.00%	0.00%	34.88%	65.12%
A link to the safety centre should be clearly visible and accessible on social media platforms	22	0.00%	0.00%	9.09%	22.73%	68.18%	43	0.00%	0.00%	0.00%	16.28%	83.72%
Information in safety centres should be available in every language and country specific resources should be included	22	0.00%	4.55%	9.09%	36.36%	50.00%	43	0.00%	0.00%	2.33%	11.63%	86.05%
Safety centres should only contain evidence-based information and resources for suicide and self-harm management and prevention	22	0.00%	0.00%	9.09%	13.64%	77.27%	43	0.00%	2.33%	0.00%	9.30%	88.37%
Safety centres should contain links to local support services	22	0.00%	0.00%	4.55%	13.64%	81.82%	43	0.00%	0.00%	2.33%	9.30%	88.37%
Companies should have in-built safety planning features and tools within their platforms	22	4.55%	22.73%	36.36%	27.27%	9.09%	43	4.65%	4.65%	11.63%	27.91%	51.16%
Companies should offer in-house brief therapeutic interventions (i.e., one session with a psychologist)	22	0.00%	27.27%	36.36%	13.64%	22.73%	43	2.33%	6.98%	6.98%	18.60%	65.12%
Companies should provide instant support such as the ability to talk to internal trained staff or trained volunteers	22	0.00%	13.64%	9.09%	45.45%	31.82%	43	0.00%	6.98%	4.65%	18.60%	69.77%
Companies should implement automatic banners on suicide and self-harm content and redirect users to helpful resources (e.g., as they did for COVID-19 posts)	22	0.00%	4.55%	18.18%	40.91%	36.36%	42	0.00%	2.38%	4.76%	23.81%	69.05%
Companies should have a function within their platforms that allows users to nominate people they would want to be contacted if in crisis	22	0.00%	9.09%	36.36%	22.73%	31.82%	43	4.65%	2.33%	6.98%	25.58%	60.47%
If a user is at risk, nominated individuals should be alerted	22	0.00%	13.64%	27.27%	27.27%	31.82%	42	2.38%	2.38%	9.52%	28.57%	57.14%
Companies should report users at risk of suicide to law enforcement/police regardless of the law in respective region	22	36.36%	22.73%	22.73%	13.64%	4.55%	43	27.91%	16.28%	18.60%	6.98%	30.23%
If a social media company has been alerted that a user is engaging in or at risk of suicide/self-harm (e.g., via a report or AI), it should send information, resources, and links to support to users via												
Email	21	9.52%	9.52%	28.57%	23.81%	28.57%	41	4.88%	12.20%	17.07%	29.27%	36.59%
Pop-up messages when searching for certain hashtags (e.g., #selfharm and known circumventing hashtags such as #selfharn)	22	0.00%	0.00%	31.82%	36.36%	31.82%	42	4.76%	0.00%	2.38%	26.19%	66.67%
Search results	20	5.00%	15.00%	15.00%	35.00%	30.00%	42	4.76%	2.38%	16.67%	38.10%	38.10%
Private/direct message	22	9.09%	4.55%	27.27%	22.73%	36.36%	42	4.76%	4.76%	16.67%	35.71%	38.10%
Their newsfeed	22	22.73%	9.09%	31.82%	27.27%	9.09%	42	4.76%	4.76%	11.90%	35.71%	42.86%
Moderating and monitoring suicide/self-harm content												
Companies should be responsible for monitoring all suicide/self-harm related content	22	0.00%	13.64%	22.73%	36.36%	27.27%	43	0.00%	9.30%	6.98%	46.51%	37.21%
Companies should moderate postings and restrict content that may be harmful to other users (e.g., graphic imagery of suicide/self-harm)	22	0.00%	9.09%	4.55%	36.36%	50.00%	43	0.00%	2.33%	0.00%	41.86%	55.81%
If a post about suicide/self-harm breaches company policies, instead of deleting it, it should be hidden from the public but remain visible to the poster	22	0.00%	18.18%	36.36%	27.27%	18.18%	43	6.98%	23.26%	18.60%	20.93%	30.23%
If a post is focused on death by suicide, the company should not allow others to comment	22	9.09%	27.27%	40.91%	13.64%	9.09%	43	18.60%	13.95%	37.21%	13.95%	16.28%
Companies should include a “downvote” function to allow users to downvote posts that are potentially harmful	22	4.55%	22.73%	40.91%	27.27%	4.55%	43	25.58%	18.60%	13.95%	20.93%	20.93%
Moderators should review and approve all suicide/self-harm content that appears in publicly available feeds prior to it being published	22	9.09%	13.64%	22.73%	27.27%	27.27%	43	4.65%	16.28%	9.30%	32.56%	37.21%
Content should be moderated such that only evidence-based strategies to cope with suicide/self-harm are publicly viewable	22	4.55%	22.73%	22.73%	31.82%	18.18%	43	4.65%	11.63%	25.58%	23.26%	34.88%
Users who encourage or promote suicide/self-harm should be reported to law enforcement	22	13.64%	22.73%	31.82%	22.73%	9.09%	42	16.67%	14.29%	21.43%	21.43%	26.19%
Social media accounts, forums and groups known to encourage suicide/self-harm should be banned	22	0.00%	4.55%	18.18%	40.91%	36.36%	42	2.38%	4.76%	2.38%	38.10%	52.38%
Companies should allow users to rate content on a “helpfulness” scale to assist with moderation	22	0.00%	0.00%	27.27%	45.45%	27.27%	43	11.63%	9.30%	16.28%	30.23%	32.56%
Companies should pause membership to their platform if a user repeatedly breaches guidelines regarding suicide/self-harm content	22	0.00%	0.00%	13.64%	36.36%	50.00%	43	4.65%	4.65%	9.30%	27.91%	53.49%
Social media companies should provide content warnings for												
All suicide/self-harm content	22	4.55%	4.55%	13.64%	54.55%	22.73%	43	0.00%	0.00%	0.00%	18.60%	81.40%
Potentially harmful suicide/self-harm content	22	4.55%	0.00%	4.55%	31.82%	59.09%	43	0.00%	0.00%	0.00%	30.23%	69.77%
Content with specific suicide/self-harm related hashtags (e.g., #suicide)	22	4.55%	0.00%	4.55%	54.55%	36.36%	43	0.00%	0.00%	2.33%	18.60%	79.07%
Specific cultures (e.g., In some Aboriginal and Torres Strait Islander cultures it is against protocol to engage with content created by or featuring peoples who have passed, particularly during periods of mourning)	22	0.00%	9.09%	27.27%	36.36%	27.27%	43	0.00%	2.33%	0.00%	23.26%	74.42%
Content warnings should include												
Helpful resources	22	0.00%	0.00%	18.18%	18.18%	63.64%	43	0.00%	0.00%	4.65%	18.60%	76.74%
The option to proceed to view content	22	0.00%	0.00%	31.82%	31.82%	36.36%	43	0.00%	0.00%	0.00%	20.93%	79.07%
Information about why there is a content warning	21	0.00%	0.00%	14.29%	33.33%	52.38%	43	0.00%	0.00%	0.00%	20.93%	79.07%
Livestreams: If a livestream of suicide/self-harm occurs, social media companies should												
Leave the livestream up for as long as possible (e.g., to allow more time for someone to intervene)	22	50.00%	13.64%	27.27%	4.55%	4.55%	43	46.51%	11.63%	27.91%	6.98%	6.98%
Leave the livestream up for the poster but make it invisible to others	22	31.82%	18.18%	27.27%	9.09%	13.64%	43	32.56%	6.98%	23.26%	25.58%	11.63%
Remove livestream content immediately	22	18.18%	13.64%	13.64%	18.18%	36.36%	43	13.95%	4.65%	23.26%	30.23%	27.91%
Remove the livestream only at the point at which a threat of suicide/self-harm turns into an attempt	22	31.82%	22.73%	45.45%	0.00%	0.00%	43	32.56%	23.26%	13.95%	20.93%	9.30%
Allow comments	22	36.36%	13.64%	18.18%	27.27%	4.55%	42	26.19%	14.29%	23.81%	23.81%	11.90%
Turn off comments	22	9.09%	22.73%	18.18%	13.64%	36.36%	43	11.63%	13.95%	30.23%	20.93%	23.26%
Send the poster resources and links to support	22	0.00%	4.55%	9.09%	13.64%	72.73%	43	0.00%	0.00%	4.65%	23.26%	72.09%
Send viewers resources and links to support immediately	22	0.00%	4.55%	0.00%	27.27%	68.18%	43	0.00%	0.00%	4.65%	18.60%	76.74%
Report the livestream to law enforcement or emergency services	22	0.00%	0.00%	9.09%	31.82%	59.09%	42	4.76%	2.38%	9.52%	14.29%	69.05%
Promoting helpful suicide/self-harm content												
Companies should promote helpful content (e.g., psychoeducation; messaging that encourages help-seeking; stories of help, hope, and recovery)	22	0.00%	4.55%	0.00%	31.82%	63.64%	43	2.33%	0.00%	2.33%	32.56%	62.79%
Companies should actively create and promote digital literacy on suicide and self-harm for all users (e.g., how to recognise and respond to suicide risk in other users)	22	0.00%	4.55%	18.18%	27.27%	50.00%	43	4.65%	0.00%	0.00%	32.56%	62.79%

#### Staffing and training

There was strong agreement that social media companies should have safety teams and paid content moderators with appropriate qualifications and experience. Moderators (and other staff who encounter self-harm and suicide-related content) should receive training and ongoing support. Companies should also provide training to influencers or content creators, on how to communicate safely about self-harm and suicide. See Table 4.

**Table 4:** Social media staffing.

Question	Professionals						Young people					
	N	Strongly Disagree %	Somewhat Disagree %	Neither Agree nor Disagree %	Somewhat Agree %	Strongly Agree %	N	Strongly Disagree %	Somewhat Disagree %	Neither Agree nor Disagree %	Somewhat Agree %	Strongly Agree %
Companies should hire mental health professionals to lead, manage, and supervise safety teams	22	0.00%	0.00%	13.64%	40.91%	45.45%	43	0.00%	0.00%	0.00%	25.58%	30.23%
Companies should invest in building safety teams with appropriate expertise in mental health	22	0.00%	0.00%	4.55%	45.45%	50.00%	43	0.00%	2.33%	0.00%	18.60%	23.26%
Companies should increase the number of moderators working at times when posting of suicide/self-harm content is more frequent (e.g., overnight, after a celebrity suicide)	22	0.00%	0.00%	13.64%	36.36%	50.00%	43	0.00%	2.33%	0.00%	23.26%	27.91%
Companies should provide support to all employees and volunteers working with suicide and self-harm content (e.g., trust and safety teams, policy teams) via specialist training, psychological support, and regular check-ins	22	0.00%	0.00%	4.55%	18.18%	77.27%	43	0.00%	0.00%	2.33%	16.28%	16.28%
Social media company moderators should be												
A paid professional who has appropriate qualifications and experience in mental health	22	0.00%	0.00%	13.64%	36.36%	50.00%	43	0.00%	0.00%	2.33%	27.91%	69.77%
Trained unpaid volunteers	22	36.36%	18.18%	31.82%	13.64%	0.00%	43	32.56%	20.93%	18.60%	18.60%	9.30%
Peers—people with living or lived experience of suicide/self-harm	22	9.09%	9.09%	36.36%	45.45%	0.00%	43	6.98%	6.98%	27.91%	46.51%	11.63%
Social media companies should provide training for moderators on												
Disclosure about suicide/self-harm	22	4.55%	0.00%	9.09%	22.73%	63.64%	43	0.00%	0.00%	0.00%	27.91%	72.09%
How to determine level of risk	22	4.55%	4.55%	13.64%	31.82%	45.45%	43	0.00%	0.00%	0.00%	23.26%	76.74%
How to respond to unsafe or reported posts related to suicide/self-harm	22	4.55%	0.00%	0.00%	22.73%	72.73%	43	0.00%	0.00%	0.00%	18.60%	81.40%
Influencers												
Companies should provide training to influencers on how they can safely communicate online about suicide/self -harm	22	0.00%	9.09%	13.64%	31.82%	45.45%	43	4.65%	0.00%	4.65%	41.86%	48.84%

### Actions policymakers should take

#### Regulation and legislation

Just over half of professionals (56.52%) and just under half of young people (48.84%) agreed that social media companies should be independently regulated by government. However, there was recognition that policy development would need to be fast tracked to keep up with the rapidly evolving social media landscape. There was strong agreement regarding the need for a special department, or regulator, to manage social media policies and that systems should be developed to appropriately monitor adherence to them.

There was lower agreement between participants regarding the type of legislation that should be developed. However, young people strongly believed that legislation should be developed prohibiting social media companies from allowing accounts that encourage or promote self-harm or suicide and that fines should be imposed for breaching this policy. They moderately agreed that social media companies should be held legally accountable for content published on their platforms. See Table 5.

**Table 5:** Regulation of social media platforms.

Question	Professionals						Young people					
	N	Strongly Disagree %	Somewhat Disagree %	Neither Agree nor Disagree %	Somewhat Agree %	Strongly Agree %	N	Strongly Disagree %	Somewhat Disagree %	Neither Agree nor Disagree %	Somewhat Agree %	Strongly Agree %
Social media companies should be independently regulated by Government	23	4.35%	4.35%	34.78%	39.13%	17.39%	43	6.98%	9.30%	34.88%	34.88%	13.95%
Government should have a system for fast tracking the development of policies relating to social media to ensure that they reflect the rapidly evolving social media landscape	23	4.35%	4.35%	17.39%	52.17%	21.74%	43	4.65%	0.00%	16.28%	53.49%	25.58%
Government should establish subcommittees or special departments who specifically develop and manage social media policies (e.g., an independent regulator for online safety such as the eSafety Commission in Australia)	23	4.35%	0.00%	8.70%	56.52%	30.43%	43	0.00%	2.33%	6.98%	55.81%	34.88%
Governments should develop legally binding safety frameworks relating to online communication of suicide / self-harm that apply to the entire social media industry	22	4.55%	4.55%	27.27%	27.27%	36.36%	43	2.33%	2.33%	16.28%	39.53%	39.53%
Government should develop systems to monitor adherence to a safety framework relating to online communication about suicide / self-harm	23	4.35%	0.00%	8.70%	52.17%	34.78%	42	2.38%	2.38%	9.52%	54.76%	30.95%
Government should create a rating system of social media companies against a set of safety standards	23	4.35%	0.00%	26.09%	43.48%	26.09%	43	2.33%	4.65%	16.28%	34.88%	41.86%
Government should use ratings of social media companies to inform the blocking of certain platforms (e.g., country wide ban, age restrictions on certain platforms) where appropriate	23	4.35%	8.70%	39.13%	30.43%	17.39%	43	4.65%	11.63%	30.23%	32.56%	20.93%
Legislation												
Policies should be developed that mandate social media companies to report to police or other emergency services when a social media user is identified as being at immediate risk of suicide (e.g., livestreams of suicide)	21	9.52%	4.76%	19.05%	47.62%	19.05%	43	2.33%	9.30%	13.95%	39.53%	34.88%
Policies should be developed that mandate social media companies to report online suicide pacts to police as soon as they are identified	21	4.76%	9.52%	28.57%	33.33%	23.81%	43	4.65%	2.33%	16.28%	32.56%	44.19%
Policies should be developed that mandate social media companies to report online suicide games to police as soon as they are identified	21	4.76%	9.52%	23.81%	33.33%	28.57%	43	2.33%	2.33%	16.28%	30.23%	48.84%
Livestreaming of a suicidal act should be illegal	21	14.29%	14.29%	42.86%	14.29%	14.29%	43	13.95%	9.30%	27.91%	23.26%	25.58%
Livestreaming of a of self-harm act (without suicidal intent) should be illegal	21	14.29%	9.52%	47.62%	14.29%	14.29%	43	16.28%	13.95%	32.56%	16.28%	20.93%
Legislation should												
Prohibit social media companies from allowing accounts or content that encourage or promote suicide / self-harm (i.e., they should remove and ban as soon as possible)	21	0.00%	14.29%	28.57%	23.81%	33.33%	43	2.33%	4.65%	9.30%	41.86%	41.86%
Hold social media companies legally accountable for the content that is published and distributed on their platforms	21	0.00%	9.52%	28.57%	42.86%	19.05%	43	0.00%	6.98%	18.60%	41.86%	32.56%
Impose fines on social media companies that allow the encouragement or promotion of suicide / self-harm	21	9.52%	0.00%	28.57%	28.57%	33.33%	43	2.33%	2.33%	9.30%	39.53%	46.51%
Impose fines on users who create content that breaches laws and company terms and conditions related to suicide / self-harm	21	4.76%	19.05%	38.10%	33.33%	4.76%	42	16.67%	19.05%	23.81%	14.29%	26.19%
All social media users should be legally mandated to report suicide / self-harm content that breaches policies / laws / guidelines / terms and conditions of use to												
Public health services or departments (e.g., an independent regulator for online safety such as eSafety Commissioner in Australia)	21	14.29%	19.05%	23.81%	33.33%	9.52%	43	13.95%	13.95%	16.28%	32.56%	23.26%
Police	21	23.81%	19.05%	33.33%	19.05%	4.76%	42	23.81%	21.43%	23.81%	14.29%	16.67%
Social media companies	21	14.29%	28.57%	33.33%	14.29%	9.52%	42	19.05%	16.67%	14.29%	33.33%	16.67%

#### Education and awareness

Table 6 shows that there was strong support for governments to require secondary schools to provide education regarding safe online communication about self-harm and suicide. There was also strong support for public education campaigns. Both groups agreed that educational programs and campaigns should be developed in partnership with young people with lived experience, mental health and suicide prevention experts, and educators.

**Table 6:** Education and awareness.

Question	Professionals						Young people					
	N	Strongly Disagree %	Somewhat Disagree %	Neither Agree nor Disagree %	Somewhat Agree %	Strongly Agree %	N	Strongly Disagree %	Somewhat Disagree %	Neither Agree nor Disagree %	Somewhat Agree %	Strongly Agree %
Government should require primary school curricula to include education about safe online communication about suicide / self-harm	22	0.00%	27.27%	27.27%	27.27%	18.18%	43	9.30%	11.63%	16.28%	27.91%	34.88%
Government should require secondary school curricula to include education about safe online communication about suicide / self-harm	22	0.00%	9.09%	18.18%	36.36%	36.36%	43	0.00%	0.00%	4.65%	27.91%	67.44%
Government should provide public education to the community about online safety regarding suicide and self-harm (e.g., via educational health promotion campaigns or resources)	22	0.00%	9.09%	18.18%	36.36%	36.36%	43	0.00%	0.00%	4.65%	27.91%	67.44%
Government should fund public health campaigns that promote safe online communication about suicide	22	0.00%	13.64%	9.09%	45.45%	31.82%	43	0.00%	0.00%	6.98%	32.56%	60.47%
Government should commission external organisations to provide public education to all citizens about online safety regarding suicide and self-harm (e.g., educational health promotion campaigns or resources like those used for tobacco (e.g., Quit) and sun cancer (e.g., SunSmart))	22	0.00%	13.64%	18.18%	36.36%	31.82%	43	0.00%	0.00%	6.98%	34.88%	58.14%
Government should develop policies and digital literacy programs regarding suicide / self-harm in partnership with												
Mental health professionals	22	0.00%	0.00%	0.00%	36.36%	63.64%	43	0.00%	0.00%	2.33%	16.28%	81.40%
Suicide prevention experts	22	0.00%	4.55%	0.00%	13.64%	81.82%	43	0.00%	0.00%	2.33%	11.63%	86.05%
Young people with living or lived experience of suicide / self-harm	22	0.00%	0.00%	0.00%	40.91%	59.09%	43	0.00%	0.00%	9.30%	23.26%	67.44%
Young people without living or lived experience of suicide / self-harm	22	4.55%	0.00%	22.73%	54.55%	18.18%	43	4.65%	25.58%	25.58%	13.95%	30.23%
Parents/guardians/carers	22	0.00%	9.09%	27.27%	40.91%	22.73%	42	2.38%	4.76%	30.95%	33.33%	28.57%
Educators and educational organisations	22	0.00%	4.55%	18.18%	40.91%	36.36%	43	2.33%	4.65%	13.95%	41.86%	37.21%

#### Collaboration and investment

Table 7 presents the views of participants on the ways they believe government should collaborate with others and future investment. Both young people and professionals agreed that governments should collaborate with their international counterparts to coordinate efforts. They agreed that an international body should be established, that an international set of safety standards be developed, and that governments should work in collaboration with the social media industry to improve online safety.

**Table 7:** Collaboration and investment.

Question	Professionals						Young people					
	N	Strongly Disagree %	Somewhat disagree %	Neither agree nor disagree %	Somewhat Agree %	Strongly Agree %	N	Strongly Disagree %	Somewhat disagree %	Neither agree nor disagree %	Somewhat Agree %	Strongly Agree %
Governments should collaborate with their international counterparts to co-ordinate efforts to prevent the promotion of suicide / self-harm online	22	0.00%	4.55%	13.64%	40.91%	40.91%	43	0.00%	0.00%	9.30%	51.16%	39.53%
An international body should be established to co-ordinate efforts to prevent suicide / self-harm online (e.g., an organisation like the International Association for Suicide Prevention or World Health Organisation)	20	5.00%	10.00%	10.00%	45.00%	30.00%	43	2.33%	2.33%	11.63%	34.88%	48.84%
An international set of safety standards related to online communication about suicide / self-harm should be developed	21	0.00%	0.00%	19.05%	38.10%	42.86%	43	2.33%	0.00%	16.28%	39.53%	41.86%
Government should work in collaboration with social media companies to improve online communication about suicide / self-harm (i.e., safer communication)	21	0.00%	0.00%	4.76%	42.86%	52.38%	43	0.00%	0.00%	11.63%	37.21%	51.16%
Government should invest in research to ensure that future guidance on safe online communication about suicide / self-harm is evidence-based	22	0.00%	0.00%	4.55%	27.27%	68.18%	43	0.00%	0.00%	13.95%	16.28%	69.77%

## Discussion

This paper reports on a cross sectional survey that examined the views of young people and suicide prevention professionals regarding the steps that both social media companies and policymakers should be taking to improve the safety of online communication about self-harm and suicide. It also sought the views of both groups of participants on some of the actions that social media companies are already taking in response to self-harm and suicide-related content.

### Key findings and implications

#### Social media companies

There was strong agreement among participants about some of the more basic measures platforms should be taking to promote safety. For example, by developing and implementing robust safety policies covering self-harm and suicide, moderating potentially harmful content, and hosting safety centres. Similarly, there was strong agreement that safety and moderation teams should be led and staffed by people with appropriate training and experience and that all staff who encounter self-harm and suicide-related content receive appropriate training and support. Although most of the major companies already employ many of these strategies there seemed to be the view that policies should be broadened, made more visible, and their implementation could be strengthened. Further, not only are many of the strategies implemented platform-specific, most have not been tested empirically for either acceptability or effectiveness. Future research should consider ways of testing content-moderation functions to ensure that they meet the needs of social media users, and consider developmental and other differences among users.

There was agreement that platforms should provide content warnings for potentially harmful content including content containing self-harm or suicide-related hashtags. As noted above, there is mixed evidence regarding the efficacy of content warnings (11, 12) and in the overarching Delphi study (23) consensus was not reached about their inclusion. However, in the study conducted by the Samaritans (11), and the current study, participants believed these would be useful, particularly if they were specific to the subject matter and contained links to helpful resources. Again, meeting the needs of social media users is important here given that participants in this study felt that trigger warnings would be helpful despite the evidence suggesting that they can at times be harmful (21). There is a need for future research to test different features and delivery of trigger warnings, specific to self-harm and suicide, that help us understand when, for whom, and in what context, these safety features are helpful.

There was less endorsement regarding how platforms should manage livestreams of self-harm or suicide. All participants agreed that livestreams should be reported to emergency services and that both posters and viewers should be sent links to helpful resources. But there was no consensus regarding how long the livestream should be allowed to run and whether or not comments should be permitted. Managing livestreams appropriately is challenging. For example, allowing the livestream to transmit for a period of time and permitting others to comment, provides an opportunity for intervention. However, it also runs the risk of viewers being exposed to a live suicide act, which would be distressing and could potentially increase the risk of others engaging in similar acts (24, 25). It also violates most platforms' policies.

There was strong agreement that platforms should use their AI capabilities to promote helpful content such as psychoeducation about self-harm and suicide, plus messaging that encourages help-seeking and stories of hope and recovery. Just as certain types of self-harm and suicide-related content can be harmful to viewers, it is well established that stories of hope and recovery can be helpful (26, 27) and there was clear support for platforms to use their capabilities to promote this. There was moderate agreement that companies should use AI to proactively detect users at risk and send them helpful information and resources. However, risk can fluctuate rapidly and there is debate in the literature regarding the accuracy of risk prediction tools in general (28, 29). There is also concern regarding some of the ways the platforms use their algorithms to direct certain types of (potentially harmful) content to (often vulnerable) users (30–32) and respondents in the current study agreed that AI should not be used for this purpose. Therefore, using AI to detect and respond to people who may be at risk will likely present ethical challenges for companies. However, studies have demonstrated that risk can be detected, with some accuracy, using content posted on platforms such as Twitter and Reddit (33, 34) and participants in the current study appeared to be relatively comfortable with the idea of companies using this type of technology if it helps keeps young people safe. Therefore, perhaps using their AI in this way could be a next step for platforms providing no harm is done in the process.

There was moderate agreement that companies provide training to influencers on how to communicate safely about self-harm and suicide to their followers. Influencers have grown in number and popularity over recent years, with many having significant numbers of (often young) followers. While some companies do provide broad mental health training to some of their content creators (35), it is important that this extends to self-harm and suicide. The new #chatsafe guidelines provide some guidance for influencers (22), and whilst this is a step in the right direction, uptake by influencers is likely to be limited. As a result, it is also important that the companies themselves support the influencers on their platforms to communicate safely online about self-harm and suicide.

There was disagreement between the groups of participants on some items. A notable example was reporting users at risk of suicide to law enforcement. Indeed, some companies have policies that state that if a user is clearly at risk of suicide, law enforcement will be contacted (36). The fact that participants did not support this (except for livestream events as discussed above) may reflect the fact that in some countries suicide remains illegal (37) and that in others, first responders may be unlikely to respond in either a timely or compassionate manner (38). It may also be seen as somewhat heavy handed and a possible breach of privacy. However, it does mean that social media companies need to think carefully about how they respond to users who are expressing acute risk on their platforms and that tailored, country-specific responses are needed.

#### Policymakers

There was strong agreement for some measures such as the need for specific departments to develop policies relating to social media and to monitor their implementation by the companies. Arguably, in Australia, we are leading the way in this regard with the creation of the e-Safety Commission which plays an important role in bridging the gap between government and the social media industry, in providing public education, and in developing and monitoring adherence to safety standards. That said, the Commission's brief is far broader than self-harm and suicide and perhaps more could be done to strengthen work in this area.

There was strong support for international collaboration and moderate support for an international body to help support online safety efforts. International collaboration on this issue makes good sense. Rates of self-harm and suicide in young people are increasing in many parts of the world (39, 40) and social media companies are multi-national, therefore, more coordinated efforts including the development of international safety standards and cross-sector collaboration is a logical next step. It's possible that international bodies such as the World Health Organisation or the International Association for Suicide Prevention could play a role here.

In terms of other steps policymakers could be taking, there was strong agreement that government should support public health campaigns promoting safe online communication. There is some evidence regarding the effectiveness of public health campaigns for suicide prevention generally (41), but to the best of our knowledge, few campaigns exist specifically on this topic or that target young people. One exception may be the #chatsafe social media campaign that was tested in two separate studies and appeared to improve young people's perceived online safety when communicating about suicide and their willingness to intervene against suicide online (12, 42). However, these were relatively small pilot studies and more work is needed to robustly assess the effectiveness of such campaigns.

There was moderate agreement that governments should require secondary school curricula to include education about safe online communication about self-harm and suicide and mixed views regarding primary schools. School curricula are already crowded, but schools are an obvious place to provide education to young people about online safety and an acceptable setting for suicide prevention activities (43). As such, it stands to reason that at least some degree of education regarding online safety when communicating online about self-harm and suicide could be a useful addition to school curricula. It may also be useful for this type of education to extend beyond students and to include both educators and parents/carers.

Somewhat surprisingly, there were mixed views regarding blanket regulation of the platforms by government, and legislation making certain types of content posted by users illegal. That said, there was some support (particularly from young people) for legislation prohibiting companies from allowing accounts that promote or encourage self-harm or suicide and that would hold companies accountable for content posted on their platforms.

### Limitations and strengths

This was by no means a large-scale representative survey; nor was it intended to be. Rather, the survey items were nested within a larger Delphi study, the main purpose of which was to inform the development of new #chatsafe guidelines. As such, the study findings cannot be generalised beyond the study population and a larger, representative survey is warranted. However, the fact that the survey was nested in the Delphi study is also a strength, as the survey was based on a robust review of the literature plus consultations with key stakeholders, including young people, policymakers, and representatives from social media companies. A further limitation was the uneven distribution of the two panels with more than twice as many young people as professionals, although the level of engagement from young people may also be considered a strength. Additionally, the youth panel comprised only young people from Australia. This was a deliberate decision to facilitate safety management during the study, but it further limits generalisability.

## Conclusions

This study examined the views of young people and suicide prevention professionals about the steps that social media companies and policymakers should take to improve online safety when it comes to self-harm and suicide. Although many of the strategies identified are already being implemented, at least to a certain extent, it is clear that more could be done.

Our findings reflect the complexity associated with trying to achieve a balance that minimises the risks of communicating online about self-harm or suicide (i.e., exposure to harmful content) whilst capitalising on some of the benefits (i.e., opportunities for intervention). Indeed, much of our work, and that of others has demonstrated that online communication about self-harm and suicide is both complex and nuanced, and content, or decisions, that may be helpful for some may be harmful for others (13, 44). With such a large user base (Meta's platforms currently have around 3.9 billion (45) users and TikTok has 755 million (46)), even small changes to their policies and practices can have a significant impact on suicide prevention efforts worldwide.

A clear message was the need for better collaboration between policymakers and the social media industry and between government and its international counterparts. To the best of our knowledge, no national or international suicide prevention policies include recommendations relating to online safety and to date there is no international body coordinating efforts in this area. In our view addressing these gaps would help to create safer online environments and would help make inroads into reducing self-harm and suicide among young people.

## Data Availability

The datasets presented in this article are not readily available because Ethical approval does not allow for sharing of data due to the vulnerable participant population. Requests to access the datasets should be directed to jo.robinson@orygen.org.au.
