# Supplementary material for: The steps that young people and suicide prevention professionals think the social media industry and policymakers should take to improve online safety. A nested cross-sectional study within a Delphi consensus approach
Source: Front Child Adolesc Psychiatry. 2023 Dec 15;2:1274263. doi: 10.3389/frcha.2023.1274263 (PMC11748789; doi:10.3389/frcha.2023.1274263)
Supplement: Supplementary file 1 [file Datasheet1.pdf]

Survey

Q77 The following items will not be included in the guidelines; however, results will be used to compile a set of supplementary recommendations for policymakers and social media companies on how they can help increase safe online communication about self-harm and suicide at a systematic rather than individual user level.

The items you will be asked to rate have been generated from peer-reviewed literature and consultations with young people, the social media industry, and policymakers.

Please note that the response options are different in this part of the survey. Please rate to what extent you agree with the following statements on a scale from 1 to 5, with 1 being strongly disagree and 5 being strongly agree.

You will have an opportunity to comment at the end of each section.

Q78 **Guidance for Social Media Companies**

To what extent do you agree with the following statements?

Q79 **Policies**

	Strongly disagree (1)	Somewhat disagree (2)	Neither agree nor disagree (3)	Somewhat agree (4)	Strongly agree (5)
Companies should provide clear policies on safe and unsafe online behaviour in relation to suicide / self-harm (1)	o	o	o	o	o
Companies should outline how they will respond to unsafe content and what actions will be taken against a user if they do not comply with safety policies (2)	o	o	o	o	o
Companies should make policies easily accessible and visible on their platforms (3)	o	o	o	o	o
If someone dies by suicide, the company should contact the next of kin to ask whether they wish to memorialise the person's profile (4)	o	o	o	o	o

Q80 Comments:

Q81 **Companies should specifically develop policies on:**

	Strongly disagree (1)	Somewhat disagree (2)	Neither agree nor disagree (3)	Somewhat agree (4)	Strongly agree (5)
Content that promotes or encourages suicide / self-harm (1)	○	○	○	○	○
Content that contains graphic descriptions or visual depictions of suicide / self-harm (2)	○	○	○	○	○
Content that details methods of, or instructions about how to engage in suicide / self-harm (3)	○	○	○	○	○
Suicide pacts, challenges, games, and hoaxes (4)	○	○	○	○	○
Mocking, doxing (publishing private and identifying information without consent), or bullying/harassing of users who have self-harmed, attempted suicide, or died by suicide (5)	○	○	○	○	○
How to report content (6)	○	○	○	○	○

Q82 **Comments:**

Q83 **Social media companies should work collaboratively with the following groups to develop policies and educational campaigns and resources:**

	Strongly disagree (1)	Somewhat disagree (2)	Neither agree nor disagree (3)	Somewhat agree (4)	Strongly agree (5)
Users regardless of living or lived experience of suicide / self-harm (i.e., young people) (1)	○	○	○	○	○
Users with lived or living experience of suicide / self-harm (2)	○	○	○	○	○
Suicide prevention experts (e.g., academic researchers) (3)	○	○	○	○	○
Mental health professionals (4)	○	○	○	○	○
Media and communications professionals (5)	○	○	○	○	○
Emergency service personnel (6)	○	○	○	○	○
Legal experts (7)	○	○	○	○	○
Parents/guardians (8)	○	○	○	○	○
Teachers (9)	○	○	○	○	○
Influencers (10)	○	○	○	○	○
Search engines (e.g, Google) (11)	○	○	○	○	○
Other social media companies (12)	○	○	○	○	○

External support services such as helplines and counselling services (13)	○	○	○	○	○
Policymakers / governments (14)	○	○	○	○	○

Q84 **Comments:**

____

Q85 **Provision of support**

	Strongly disagree (1)	Somewhat disagree (2)	Neither agree nor disagree (3)	Somewhat agree (4)	Strongly agree (5)
All companies should have a safety centre (1)	o	o	o	o	o
A link to the safety centre should be clearly visible and accessible on social media platforms (2)	o	o	o	o	o
Information in safety centres should be available in every language and country specific resources should be included (3)	o	o	o	o	o
Safety centres should only contain evidence-based information and resources for suicide and self-harm management and prevention (4)	o	o	o	o	o
Safety centres should contain links to localised support services organised by the support they offer and the population of interest (e.g., helplines) (5)	o	o	o	o	o
Companies should offer in-house brief therapeutic interventions (i.e., one session with a psychologist) (6)	o	o	o	o	o

Companies should provide instant support such as the ability to talk to internal trained staff or volunteers who have specialist training in responding to suicide / self-harm (7)	○	○	○	○	○
Companies should implement automatic banners on suicide and self-harm content and redirect users to helpful resources (e.g., as they did for COVID-19 posts) (8)	○	○	○	○	○
Companies should have in-built safety planning features and tools within their platforms (9)	○	○	○	○	○
Companies should have a function within their platforms (e.g., in settings) that allows users to nominate people who they would want to be contacted during an emergency or if they need support such as suicide risk (10)	○	○	○	○	○
If a user is at risk, nominated individuals should be alerted (11)	○	○	○	○	○

Companies should report users at risk of suicide to law enforcement/police regardless of the law in respective region (12)	○	○	○	○	○

Q86 **Comments:**

____

____

____

____

____

Q87 **If a social media company has been altered that a user is engaging in or at risk of suicide / self-harm (e.g., via a report or artificial intelligence), it should send information, resources, and links to support to users via:**

	Strongly disagree (1)	Somewhat disagree (2)	Neither agree nor disagree (3)	Somewhat agree (4)	Strongly agree (5)
Email (1)	○	○	○	○	○
Pop-up messages when searching for certain hashtags (e.g., #selfharm and known circumventing hashtags such as #selfharn) (2)	○	○	○	○	○
Search results (3)	○	○	○	○	○
Private / direct message (4)	○	○	○	○	○
Their feed (5)	○	○	○	○	○

Q88 **Comments:**

____

____

____

____

____

Q89 **Artificial intelligence and algorithms**

	Strongly disagree (1)	Somewhat disagree (2)	Neither agree nor disagree (3)	Somewhat agree (4)	Strongly agree (5)
Companies should utilise artificial intelligence (AI) to identify harmful mainstream media coverage of suicide / self-harm (1)	○	○	○	○	○
Companies should utilise AI to identify users indicating risk of suicide / self-harm (2)	○	○	○	○	○
Companies should use AI to send helpful information, resources, and links to support to users at risk (3)	○	○	○	○	○
Companies should not use AI to detect risk and intervene in any way because it is unethical and should not be done (4)	○	○	○	○	○

Companies should redirect search results for suicide / self-harm to more helpful content (e.g., content designed to educate or instill hope) (5)	○	○	○	○	○
Companies should remove autocomplete searches for terms relating to suicide and self-harm methods (6)	○	○	○	○	○
Companies should not allow harmful suicide / self-harm content to appear in 'suggested content' (7)	○	○	○	○	○

Q90 **Comments:**

____

____

____

____

____

Q91 **Moderating and monitoring suicide / self-harm content**

	Strongly disagree (1)	Somewhat disagree (2)	Neither agree nor disagree (3)	Somewhat agree (4)	Strongly agree (5)
Companies should be responsible for monitoring all suicide / self-harm related content (1)	○	○	○	○	○
Companies should moderate postings and restrict content that may be harmful to other users (e.g., graphic imagery of suicide / self-harm) (2)	○	○	○	○	○
If a post about suicide / self-harm breaches company policies, instead of deleting the post, the company should hide it from the public but keep the post visible to the poster (3)	○	○	○	○	○

If a post is focused on death by suicide, the company should not allow others to comment (4)	○	○	○	○	○
Companies should include a ‘downvote’ function to allow users to downvote posts that are potentially harmful (5)	○	○	○	○	○
Moderators should review and approve all suicide / self-harm content that appears in publicly available feeds prior to it being published (6)	○	○	○	○	○
Content should be moderated such that only evidence-based strategies to cope with suicide / self-harm are publicly viewable (7)	○	○	○	○	○

Users who encourage or promote suicide / self should be reported to law enforcement (8)	○	○	○	○	○
Social media accounts, forums and groups known to encourage suicide / self-harm should be banned (9)	○	○	○	○	○
Companies should allow users to rate content on a ‘helpfulness’ scale to assist with moderation (10)	○	○	○	○	○
Companies should pause memberships to their platform if a user repeatedly posts content that breaches user guidelines regarding suicide / self-harm content (11)	○	○	○	○	○

Q92 **Comments:**

Q93 **Social media companies should provide content warnings for:**

	Strongly disagree (1)	Somewhat disagree (2)	Neither agree nor disagree (3)	Somewhat agree (4)	Strongly agree (5)
All suicide / self-harm content (1)	○	○	○	○	○
Potentially harmful suicide / self-harm content (2)	○	○	○	○	○
Content with specific suicide / self-harm related hashtags (e.g., #suicide) (3)	○	○	○	○	○
Specific cultures (e.g., In some Aboriginal and Torres Strait Islander cultures it is against protocol to engage with content created by or featuring peoples who have passed, particularly during periods of mourning) (4)	○	○	○	○	○

Q94 **Comments:**

____

Q95 **Content warnings should include:**

	Strongly disagree (1)	Somewhat disagree (2)	Neither agree nor disagree (3)	Somewhat agree (4)	Strongly agree (5)
Helpful resources (1)	○	○	○	○	○
The option to proceed to view content (2)	○	○	○	○	○
Information about why there is a content warning in place (3)	○	○	○	○	○

Q96 **Comments:**

____

____

____

____

____

Q97 **Social media companies should provide users with step-by-step information on:**

	Strongly disagree (1)	Somewhat disagree (2)	Neither agree nor disagree (3)	Somewhat agree (4)	Strongly agree (5)
How to report harmful/ unsafe suicide / self-harm content to the platform (1)	○	○	○	○	○
What happens after content is reported (2)	○	○	○	○	○
If the user will be notified of who reported them (3)	○	○	○	○	○
What is communicated to the user who was reported (4)	○	○	○	○	○

Q98 **Comments:**

____

____

____

____

____

Q99 **Reporting**

	Strongly disagree (1)	Somewhat disagree (2)	Neither agree nor disagree (3)	Somewhat agree (4)	Strongly agree (5)
Companies should promote a culture of reporting and reduce stigma around this (1)	○	○	○	○	○
Companies should review all suicide or self-harm related reports (2)	○	○	○	○	○
Companies should prioritise user reports based on level of risk (3)	○	○	○	○	○
Moderators should inform users about why their content has been removed (4)	○	○	○	○	○
Companies should expand reporting categories to cover a broader range of unsafe behaviour online including suicide / self-harm (5)	○	○	○	○	○

Companies should keep users who submit reports informed as to the general progress and actions taken because of the report. If you agree, with this statement, what should the timeframe be (e.g., 24 hours, one week etc.)? (6)	○	○	○	○	○

Q100 **Comments:**

____

____

____

____

____

Q101 **Promoting helpful suicide / self-harm content**

	Strongly disagree (1)	Somewhat disagree (2)	Neither agree nor disagree (3)	Somewhat agree (4)	Strongly agree (5)
Companies should promote helpful content (e.g., psychoeducation; messaging that encourages help-seeking; stories of help, hope, and recovery) (1)	o	o	o	o	o
Companies should actively create and promote digital literacy on suicide and self-harm for all users (e.g., how to recognise and respond to suicide risk in other users) (2)	o	o	o	o	o

Q102 **Comments:**

____

____

____

____

____

Q103 **Influencers**

	Strongly disagree (1)	Somewhat disagree (2)	Neither agree nor disagree (3)	Somewhat agree (4)	Strongly agree (5)
Companies should provide training to influencers on how they can safely communicate online about suicide / self - harm (1)	○	○	○	○	○

Q104 **Comments:**

____

____

____

____

____

Q105 **Privacy and agency**

	Strongly disagree (1)	Somewhat disagree (2)	Neither agree nor disagree (3)	Somewhat agree (4)	Strongly agree (5)
Companies should verify the identity of all users (1)	○	○	○	○	○
Companies should encourage users to restrict their online network to people they know (2)	○	○	○	○	○
Companies should maximise user agency by building in functions that enable users to filter specific types of content and decide what they want to see and what they do not want to see (e.g., suicide, self-harm etc.) (3)	○	○	○	○	○

Q106 **Comments:**

____

____

____

Q107 **Responsibility**

	Strongly disagree (1)	Somewhat disagree (2)	Neither agree nor disagree (3)	Somewhat agree (4)	Strongly agree (5)
Companies should not be responsible for the safety of underage users (>18 years). The duty of care for underage users should lie with parents/guardians (1)	○	○	○	○	○
Companies are responsible for the content that is published in on their platforms (2)	○	○	○	○	○
Responsibility for content should be held by the user not the company (3)	○	○	○	○	○
Companies and policymakers are both responsible for safety; however, the platform should own the bulk of the responsibility for safety of users (4)	○	○	○	○	○
Companies should restrict underage users (>18 years) from exposure to suicide / self-harm content (5)	○	○	○	○	○

Q108 **Comments:**

Q109 Social media companies **should:**

	Remove (i.e, take down/delete) (1)	Shadow ban (i.e.,remove from public view. The poster will still see the post) (2)	Allow users to view content, but disable interactions (i.e., users can see the post but cannot share, comment, like or react) (3)	Restrict (e.g., add an age restriction, content warning, or blur) (4)	Unsure (5)
Any images depicting suicide / self-ham (1)	○	○	○	○	○
Images of new suicide / self-harm wounds and injuries (2)	○	○	○	○	○
Images of healed suicide / self-harm injuries (3)	○	○	○	○	○
Artistic suicide / self-harm images (e.g., tattoos) (4)	○	○	○	○	○
Images of suicide / self-harm methods and locations (5)	○	○	○	○	○
Hashtags related to suicide / self-harm (6)	○	○	○	○	○
Livestreams of suicide / self-harm (7)	○	○	○	○	○
Responses to livestreams of suicide / self-harm (8)	○	○	○	○	○

Videos of the lead up to or process of suicide (9)	○	○	○	○	○
Videos of suicide rescue footage (10)	○	○	○	○	○
Graphic descriptions of self-harm / suicide (11)	○	○	○	○	○
Suicide notes (12)	○	○	○	○	○
Fictional suicide / self-harm content (e.g., illustrations, animations, memes, video games) (13)	○	○	○	○	○
Suicide hoaxes (14)	○	○	○	○	○
Suicide games and challenges (15)	○	○	○	○	○
Educational suicide / self-harm content (16)	○	○	○	○	○
Misinformation about suicide / self-harm (17)	○	○	○	○	○
Content that identifies and shares details of those who have engaged in suicide / self-harm (doxing) (18)	○	○	○	○	○

Accounts dedicated to suicide / self-harm of any nature (19)	○	○	○	○	○
Accounts that are pro-suicide / self-harm (20)	○	○	○	○	○
Accounts that support understanding, reduction, cessation of suicide / self-harm (21)	○	○	○	○	○

Q110 **Comments:**

____

____

____

____

____

Q111 If a livestream of suicide / self-harm occurs, social media companies **should:**

	Strongly disagree (1)	Somewhat disagree (2)	Neither agree nor disagree (3)	Somewhat agree (4)	Strongly agree (5)
Leave the livestream up for as long as possible (e.g., to allow more time for someone to intervene) (1)	○	○	○	○	○
Leave the livesteam up for the poster but make it invisible to others (2)	○	○	○	○	○
Remove livestream content immediately (3)	○	○	○	○	○
Remove the livestream only at the point at which a threat of suicide / self-harm turns into an attempt (4)	○	○	○	○	○
Allow comments (5)	○	○	○	○	○
Turn off comments (6)	○	○	○	○	○
Send the poster resources and links to support (7)	○	○	○	○	○

Send viewers resources and links to support immediately (8)	○	○	○	○	○
Report the livestream to law enforcement or emergency services (9)	○	○	○	○	○

Q112 **Comments:**

____

____

____

____

____

Q113 **Staff**

	Strongly disagree (1)	Somewhat disagree (2)	Neither agree nor disagree (3)	Somewhat agree (4)	Strongly agree (5)
Companies should hire mental health professionals such as clinical psychologists to lead, manage, and supervise safety teams (1)	○	○	○	○	○
Companies should invest in building safety teams with appropriate expertise in mental health (e.g., allied health professionals such as psychologists, social workers, youth workers, occupational therapists etc.) (2)	○	○	○	○	○

Companies should increase the number of moderators working at specific times at which suicide / self-harm content is known to be higher (e.g., overnight, after a celebrity suicide) (3)	○	○	○	○	○
Companies should provide support to all employees and volunteers working with suicide and self-harm content (e.g., trust and safety teams, policy teams) via specialist training, psychological support, and regular managerial check-ins (4)	○	○	○	○	○

Q114 **Comments:**

____

____

____

Q115 **Social media company moderators of should be:**

	Strongly disagree (1)	Somewhat disagree (2)	Neither agree nor disagree (3)	Somewhat agree (4)	Strongly agree (5)
A paid professional who has appropriate qualifications and experience in mental health (1)	o	o	o	o	o
A paid professional who does not have mental health qualifications or experience (2)	o	o	o	o	o
Trained unpaid volunteers (3)	o	o	o	o	o
Peers - people with living or lived experience of suicide / self-harm (4)	o	o	o	o	o

Q116 **Comments:**

____

____

____

____

____

Q117 **Social media companies should provide guidance and training for moderators on:**

	Strongly disagree (1)	Somewhat disagree (2)	Neither agree nor disagree (3)	Somewhat agree (4)	Strongly agree (5)
Self-disclosure about suicide / self-harm (1)	o	o	o	o	o
How to determine level of risk (2)	o	o	o	o	o
How to respond to unsafe or reported posts related to suicide / self-harm (3)	o	o	o	o	o

Q118 **Comments:**

____

____

____

____

____

End of Block: Additional 1

Start of Block: Additional 2

Q119 **Guidance for policy and policymakers**

To what extent do you agree with the following statements?

Q120 **Regulation**

	Strongly disagree (1)	Somewhat disagree (2)	Neither agree nor disagree (3)	Somewhat agree (4)	Strongly agree (5)
Social media companies should be independently regulated by Government (1)	○	○	○	○	○
Government should have a system for fast tracking the development of policies relating to social media to ensure that they reflect the rapidly evolving social media landscape (2)	○	○	○	○	○
Government should establish subcommittees or special departments who specifically develop and manage social media policies (e.g., an independent regulator for online safety such as the eSafety Commissioner in Australia) (3)	○	○	○	○	○

Governments should develop legally binding safety frameworks relating to online communication of suicide / self-harm that apply to the entire social media industry (i.e., all social media companies where applicable) (4)	○	○	○	○	○
Government should develop systems that enable them to monitor adherence to a safety framework relating to online communication about suicide / self-harm (5)	○	○	○	○	○
Government should create a rating system of social media companies against a set of safety standards (6)	○	○	○	○	○

Government should use ratings of social media companies to inform the blocking of certain platforms (e.g., country wide ban, age restrictions on certain platforms) where appropriate (7)	○	○	○	○	○

Q121 **Comments:**

____

____

____

____

____

Q122 **Legislation**

	Strongly disagree (1)	Somewhat disagree (2)	Neither agree nor disagree (3)	Somewhat agree (4)	Strongly agree (5)
Policies should be developed that mandate social media companies to report to police or other emergency services when a social media user is identified as being at immediate risk of suicide (e.g., livestreams of suicide) (1)	○	○	○	○	○
Policies should be developed that mandate social media companies to report online suicide pacts to police as soon as they are identified (2)	○	○	○	○	○
Policies should be developed that mandate social media companies to report online suicide games to police as soon as they are identified (3)	○	○	○	○	○

Livestreaming of a suicidal act should be illegal (4)	○	○	○	○	○
Livestreaming of a of self-harm act (without suicidal intent) should be illegal (5)	○	○	○	○	○

Q123 **Comments:**

____

____

____

____

____

Q124 **Legislation should:**

	Strongly disagree (1)	Somewhat disagree (2)	Neither agree nor disagree (3)	Somewhat agree (4)	Strongly agree (5)
Prohibit social media companies from allowing accounts or content that encourage or promote suicide / self-harm (i.e., they should remove and ban as soon as possible) (1)	○	○	○	○	○
Hold social media companies legally accountable for the content that is published and distributed on their platforms (2)	○	○	○	○	○
Impose fines on social media companies that allow the encouragement or promotion of suicide / self-harm (3)	○	○	○	○	○
Impose fines on users who create content that breaches laws and company terms and conditions related to suicide / self-harm (4)	○	○	○	○	○

Q125 **Comments:**

____

____

____

____

____

Q126 **All social media users should be legally mandated to report suicide / self-harm content that breaches policies / laws / guidelines / terms and conditions of use to:**

	Strongly disagree (1)	Somewhat disagree (2)	Neither agree nor disagree (3)	Somewhat agree (4)	Strongly agree (5)
Public health services or departments (e.g., an independent regulator for online safety such as eSafety Commissioner in Australia) (1)	○	○	○	○	○
Police (2)	○	○	○	○	○
Social media companies (3)	○	○	○	○	○

Q127 **Comments:**

____

Q128 **Education**

	Strongly disagree (1)	Somewhat disagree (2)	Neither agree nor disagree (3)	Somewhat agree (4)	Strongly agree (5)
Government should require primary school curricula to include education about safe online communication about suicide / self-harm (1)	○	○	○	○	○
Government should require secondary school curricula to include education about safe online communication about suicide / self-harm (2)	○	○	○	○	○
Government should provide public education to all their citizens about online safety regarding suicide and self-harm (e.g., educational health promotion campaigns or resources) (3)	○	○	○	○	○

Government should commission other external organisations to provide public education to all citizens about online safety regarding suicide and self-harm (e.g., educational health promotion campaigns or resources like those used for tobacco (e.g., Quit) and sun cancer (e.g., SunSmart)) (4)	○	○	○	○	○

Q129 **Comments:**

____

____

____

____

____

Q130 **Government should develop policies and digital literacy programs regarding suicide / self-harm with:**

	Strongly disagree (1)	Somewhat disagree (2)	Neither agree nor disagree (3)	Somewhat agree (4)	Strongly agree (5)
Mental health professionals (1)	○	○	○	○	○
Suicide prevention experts (2)	○	○	○	○	○
Young people with living or lived experience of suicide / self-harm (3)	○	○	○	○	○
Young people without living or lived experience of suicide / self-harm (4)	○	○	○	○	○
Parents/guardians/carers (5)	○	○	○	○	○
Educators and educational organisations (6)	○	○	○	○	○
Celebrities / influencers with lived experience (7)	○	○	○	○	○
Celebrities / influencers without lived experience (8)	○	○	○	○	○

Q131 **Comments:**

____

____

____

____

____

Q132 **Collaboration**

	Strongly disagree (1)	Somewhat disagree (2)	Neither agree nor disagree (3)	Somewhat agree (4)	Strongly agree (5)
Governments should collaborate with their international counterparts to co-ordinate efforts to prevent the promotion of suicide / self-harm online (1)	○	○	○	○	○
An international body should be established to co-ordinate efforts to prevent suicide / self-harm online (e.g., an organisation like the International Association for Suicide Prevention or World Health Organisation) (2)	○	○	○	○	○
An international set of safety standards related to online communication about suicide / self-harm should be developed (3)	○	○	○	○	○

Government should work in collaboration with social media companies to improve online communication about suicide / self-harm (i.e., safer communication) (4)	o	o	o	o	o

Q133 **Comments:**

____

____

____

____

____

Q134 **Investment**

	Strongly disagree (1)	Somewhat disagree (2)	Neither agree nor disagree (3)	Somewhat agree (4)	Strongly agree (5)
Government should fund research to ensure that future guidance on safe online communication about suicide / self-harm is evidence-based (1)	○	○	○	○	○
Government should fund public health campaigns that promote safe online communication about suicide (2)	○	○	○	○	○

Q135 **Comments:**

____

____

____

____

____
